# Fecal Microbiota Transplantation Can Alleviate Gastrointestinal Transit in Rats with High-Fat Diet-Induced Obesity via Regulation of Serotonin Biosynthesis

**DOI:** 10.1155/2018/8308671

**Published:** 2018-10-02

**Authors:** Wenjing Sun, Yan Guo, Shirong Zhang, Zhihui Chen, Kangqi Wu, Qin Liu, Kaijun Liu, Liangzhi Wen, Yanling Wei, Bin Wang, Dongfeng Chen

**Affiliations:** Department of Gastroenterology, Research Institute of Surgery, Daping Hospital, Army Medical University, Chongqing 400042, China

## Abstract

**Aim:**

We tested the hypothesis that fecal microbiota transplantation (FMT) could regulate the biotransformation of bile acids, such as deoxycholic acid (DCA) and cholic acid (CA), which in turn regulate the biosynthesis of serotonin in the gut and relieve gastrointestinal dysmotility in high-fat diet- (HFD-) induced obesity in rats.

**Methods:**

Male Sprague-Dawley rats were randomly divided into the control diet group, HFD group, and HFD-fed with receiving FMT. HFD was fed for 12 weeks. At the end of two-week HFD, FMT was carried out for two weeks. The gastrointestinal transit, serotonin concentration, the expression of tryptophan hydroxylase 1 (TPH1) and serotonin reuptake transporter (SERT), and the levels of bile acids in intestinal contents were examined.

**Results:**

Compared with the control group, the gastrointestinal transit and small intestinal serotonin concentration of HFD-fed rats were increased. In HFD-fed rats, TPH1 protein expression was increased significantly, while SERT protein expression was decreased, but not significant. The levels of CA and DCA in intestinal contents were also significantly increased in HFD-fed rats compared with the control group. After HFD-fed rats receiving FMT treatment, the gastrointestinal transit, small intestinal serotonin concentration, and TPH1 expression were decreased, while SERT expression was not affected. Moreover, the levels of CA and DCA in intestinal contents were also decreased.

**Conclusions:**

FMT could alleviate small intestinal transit in the HFD-fed rats by regulating the serotonin biosynthesis. In this process, CA and DCA may be related to the regulation of synthesis of serotonin.

## 1. Introduction

Obesity is an increasingly epidemic problem in most countries in the world and induces various systemic diseases including heart disease, nonalcoholic fatty liver disease (NAFLD), type 2 diabetes, colorectal cancer, and metabolic syndrome [1]. Besides, recent studies have shown that obesity and high-fat diet (HFD) are associated with the gastrointestinal dysmotility [2]. Higher BMI and obesity are related to diarrhea [3, 4]. Another study indicated that 19.2% of obese person has diarrhea [5]. On the other hand, orocecal transit time in obese children was faster compared with that in nonobese children [6]. In addition, increased in small bowel contractility and a more rapid intestinal transit are often observed in adult obese patients [7–9]. These abnormal gastrointestinal motilities can affect the rate of digestion and appetite to induce or inhibit hunger [2, 10]. Hence, obesity is likely to impair physiological function of gut and lower quality of life and social function finally. Unfortunately, effective treatments are lacking so far [11].

However, the etiology of the association between obesity and gastrointestinal dysmotility are not clearly understood. Previous studies showed that the factors affecting gastrointestinal motility in obesity include several pathophysiological factors, of which neurotransmitters, gut microbiota, and microbiota-derived metabolites have been proposed as the potentially important factors [2]. Recent evidence suggests that obesity is associated with increased levels of serotonin, also known as 5-hydroxytryptamine (5-HT), in the gut [12], and inhibiting serotonin could reduce obesity and its related disorders [13]. It is well-known that serotonin, which can regulate gastrointestinal motility, is synthesized from tryptophan in enterochromaffin (EC) cells of the gastrointestinal tract by the rate-limiting enzyme, tryptophan hydroxylase 1 (TPH1); it is then stored in secretory granules until its release into the lumen or lamina propria when the EC cells are activated by chemical or mechanical stimulation [14]. The extracellular 5-HT levels are predominately regulated by the serotonin transporter (SERT) localized on enterocytes near the EC cells; 5-HT is then metabolized by monoamine oxidase A to 5-hydroxyindoleacetic acid [14, 15]. Therefore, drugs targeting on the serotonin system, such as serotonin receptor agonist and antagonists, are used in the treatment of motility disorders. Although these drugs can relieve the patients' symptoms, there are also some dangerous adverse effects, such as arrhythmia [16, 17]. Thus, it is crucial to find a new therapeutic approach to relieve gastrointestinal dysmotility associated with obesity.

Recently, an increasing number of studies have indicated that the gut microbiota plays a vital role in regulating gastrointestinal motility [18, 19]. The gut microbiota of patients with irritable bowel syndrome with diarrhea (IBS-D), which is characterized by increased gastrointestinal motility together with diarrhea and abdominal pain, is different from that of individuals with a healthy gut [20]. It was reported that the proportion of Firmicutes and Bacteroidetes in IBS patients is different. Tap et al. [21] reported that the gut microbiota of IBS-D patients was associated with an enriched Bacteroides enterotype. In addition, gut microbiota and gut microbial products could regulate gastrointestinal motility via serotonin synthesis [22]. Notably, in the regulation of serotonin synthesis, bile acids, such as deoxycholic acid (DCA), which are produced by microbial biotransformation of cholic acid (CA) secreted by the liver [23], play an important role. DCA and CA could upregulate the expression of TPH1, which would lead to an increase in serotonin. Meanwhile, gut microbiota is also associated with the biotransformation of bile acids [24]. Thus, these pathophysiological changes and the interactions between gut microbiota and serotonin synthesis demonstrated that gut microbiota and microbial metabolites could affect the biosynthesis of serotonin and lead to changes in gastrointestinal motility.

Presently, fecal microbiota transplantation (FMT), which is recognized as a potential therapy to change the gut microbiota, can alleviate gastrointestinal dysmotility, IBS, inflammatory bowel diseases, chronic gastrointestinal infections, obesity, and metabolic syndrome-related diseases [25, 26]. However, the potential mechanisms underlying FMT for treatment of gastrointestinal dysmotility in obese persons have not been completely elucidated, even in the animal models. Thus, we hypothesized that FMT could regulate the biotransformation of bile acids, such as DCA and CA, which in turn regulate the biosynthesis of serotonin in the gut and relieve gastrointestinal dysmotility. This could lead to the alleviation of gastrointestinal transit in high-fat diet-induced obesity in rats.

## 2. Material and Methods

### 2.1. Animal Experiments, Diets, and Small Intestinal Motility Assay

**Figure 1:**
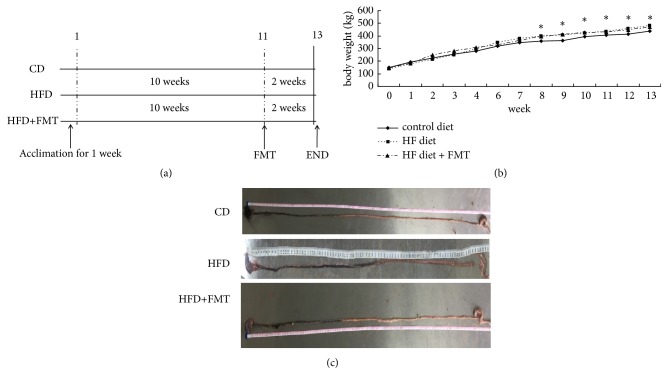
FMT alleviates the gastrointestinal transit in HFD-fed rats. (a) The flow chart of the experimental design. 30 male rats were randomly divided into the three groups (10 rats per group). (b) Body weight of the three group. (c) The small intestine and the gastrointestinal transit assay. Data were expressed as mean ± SEM (n=3~10), *∗* p<0.05 compared with the control group.

Sprague-Dawley rats (age, four weeks; weight, 100 ± 10 g; male) were purchased from and housed in the Experimental Animal Center of the Research Institute of Surgery, Daping Hospital, Army Medical University (Chongqing, China). All experimental protocols were approved by the Animal Experimentation Ethics Committee of the Army Medical University. All rats were fed at 22 ± 2°C, 55 ± 10% humidity, under a 12-h light/dark cycle. All rats were allowed access to food and water freely. After one week of acclimation, a total of 30 rats were divided into three groups (10 rats per group) randomly. The control group was raised on standard diet (10% kcal fat, diet formula D12450B) for 12 weeks. The HFD group and the HFD + FMT group were raised on an HFD (45% kcal fat, diet formula D12451, Research Diets, New Brunswick, NJ) for 12 weeks [27]. From the 11th week to the 13th week, the HFD + FMT group was subjected to FMT (Figure 1(a)). In this treatment, 4 g of fresh fecal samples were collected from the control group once upon defecation and homogenized in 20 ml of 0.9% NaCl for 3 min. Then, 2 ml of the settled suspension was gavaged in the HFD + FMT group rats. At the same time, 2 ml of 0.9% NaCl was gavaged to the HFD-fed rats and the control group. Body weight was recorded once a week. At the 13th week, fresh fecal samples of each rat were collected and stored at −80°C. Small intestinal motility was assessed by the charcoal test [28], where 2 ml of charcoal solution (0.5 g charcoal, 0.25 g gum Arabic, and 5 ml 0.9% NaCl) was administered intragastrically by gavage. After 10 min, the rats were sacrificed, and the small intestine was removed gently. The migration of the charcoal solution from the pylorus along the small intestine was measured with a ruler [28, 29]. Intestinal transit for each rat was calculated with the following formula: Intestinal transit (%) = (the distance traveled by the test liquid / the total length of intestine) × 100% [29, 30]. While the rats were sacrificed, peripheral blood was collected and stored at −20°C to assess the concentration of serotonin. The small intestine and colon tissues were either fixed in 4% paraformaldehyde solution or stored at −80°C.

### 2.2. Measurement of the Serotonin Concentration in the Serum, Small Intestine, and Colon

The frozen small intestine and colon tissue were homogenized in 0.05 M HCl and 0.1% ascorbic acid, respectively [31]. The serotonin concentration in the serum, small intestine, and colon tissue (in ng/mg weight) was determined using an enzyme-linked immunosorbent assay kit (sensitivity 0.293 ng/ml) (ADI-900-175, Enzo Life Sciences, Switzerland). The serotonin concentration from the tissue samples was normalized to the total protein content as detected by bicinchoninic acid (BCA) assay (BioTeke Corporation, Beijing, China).

### 2.3. Immunohistochemical Staining of Serotonin

The small intestine specimens were embedded in paraffin, cut into serial 5-*μ*m sections, and mounted on slides. The sections were deparaffinized and rehydrated in xylene and ethanol. Antiserotonin antibody was added (S5545, Sigma-Aldrich). Biotin-Streptavidin/peroxidase-conjugated secondary antibody (SP-900, ZSGB-Bio, Beijing, China) was used, and the reaction was visualized with 3,3′-diaminobenzidine tetrahydrochloride as the chromogen. The slides were counterstained with hematoxylin. The positive (stained) areas were quantified with the Image J software (National Institutes of Health, USA).

### 2.4. Western Blotting

The entire small intestine samples (2 cm regions of the proximal, medial, and distal of the rat small intestine) were homogenized in lysis buffer with 1 mM phenylmethylsulfonyl fluoride and centrifuged at 15,000 × g for 15 min. The protein concentrations were measured by the BCA assay (BioTeke Corporation, Beijing, China). After denaturation, proteins were subjected to SDS-PAGE, blotted onto polyvinylidene difluoride membranes and blocked in Tris-buffered saline containing 0.1% Tween-20 with 5% nonfat milk for 2 h. Then, the membranes were incubated with antibody against TPH1 (1:500) (ab52954, Abcam), SERT (1:250) (ARG63804, Arigo) or glyceraldehyde 3- phosphate dehydrogenase (GAPDH) (1:1000) (TA802519, Origen) overnight at 4°C. After overnight incubation, the membranes were washed in Tris-buffered saline containing 0.1% Tween-20 thrice and incubated with anti-rabbit IgG (1:10000) (ZB-5301, ZSGB-BIO, Beijing, China), anti-goat IgG (1:15000) (ZB-2306, ZSGB-BIO, Beijing, China), and anti-mouse IgG (1:10000) (ATA0011, ATgene Biotech, Chongqing, China) for 2 h each and then visualized by electrochemiluminescence detection (Fujifilm, Japan).

### 2.5. Bile Acid Quantification of the Intestinal Contents

High pressure liquid chromatography (HPLC) (Waters, USA) was performed to measure the level of bile acids. Standard solutions of CA and DCA (Aladdin, Shanghai, China) at various concentrations (1–100 ng/ml) were prepared. The intestinal contents samples were dissolved in 50% methanol (50 mg intestinal contents samples and 200 *μ*g 50% methanol) [32] and then analyzed by HPLC to measure the concentration of CA and DCA.

### 2.6. Statistical Analyses

Data were represented as mean ± standard error of the mean (SEM). Statistical comparison between the three groups was performed using the analysis of variance (ANOVA) and Student's t-tests. For qRT-PCR and Western blotting, TPH1 and SERT levels were normalized to those of GAPDH, respectively. A P-value < 0.05 was considered statistically significant. The statistical analyses were performed using SPSS 22.0 (SPSS, Chicago, IL, USA) software.

## 3. Results

### 3.1. Evaluation of the Body Weight, Liver Weight, and Intestinal Transit

HFD-fed rats tended to have a higher body weight. The average body weight of the HFD-fed rats was higher compared with that of the rats in the control group (Figure 1(b)). The HFD-fed rats also had a higher liver weight and the ratio of liver to body weight (P < 0.001, Table 1). After FMT for two weeks, the body weight, liver weight, and the ratio of liver to body weight in the HFD-fed rats were decreased compared with the nontreated HFD-fed rats (Table 1). In the intestinal transit analysis, there was no significant difference in the lengths of the small intestine for the three groups of rats (Table 1). Gastrointestinal transit was faster in the HFD-fed rats than in the standard diet-fed rats. After FMT for two weeks, the gastrointestinal transit was slower in the HFD-fed FMT rats than in rats without FMT treatment (Table 1).

### 3.2. Analysis of the Intestinal Serotonin System in HFD Rats

**Figure 2:**
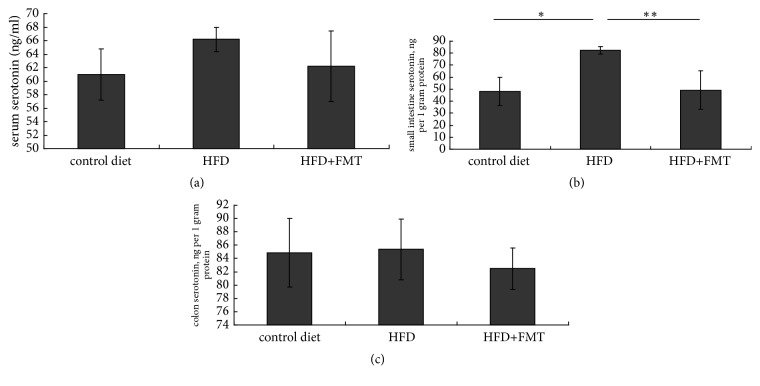
The concentration of serotonin in serum, small intestine, and colon among the three groups (n=6~8). *∗* p=0.020, compared with rats fed by control diet; *∗∗* p=0.030, compared with HFD-fed rats receiving FMT.

**Figure 3:**
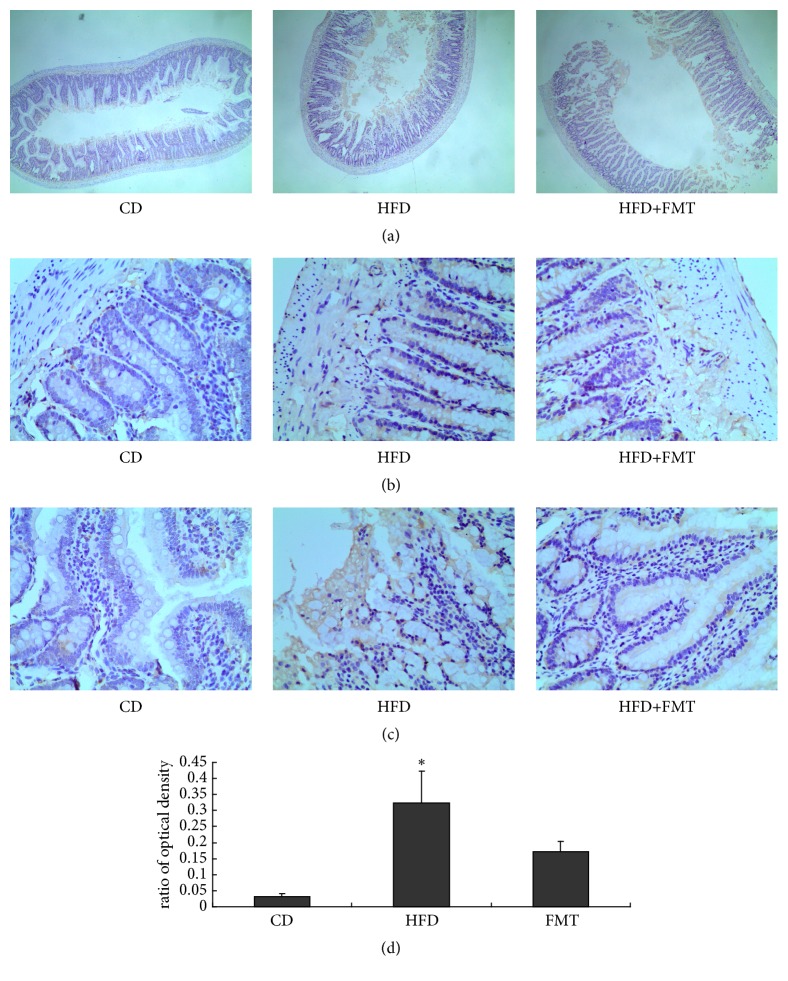
High-fat diets increased the levels of serotonin in small intestine. (a) Serotonin staining of small intestine in the three groups (×4). (b), (c) Brown areas show serotonin positive (×40). (d) The areas of serotonin positive in small intestine were measured in randomly selected fields from each slide. Statistical graph of quantified optical density is shown in (d). *∗*p=0.012, compared with the control diet-fed rats.

**Figure 4:**
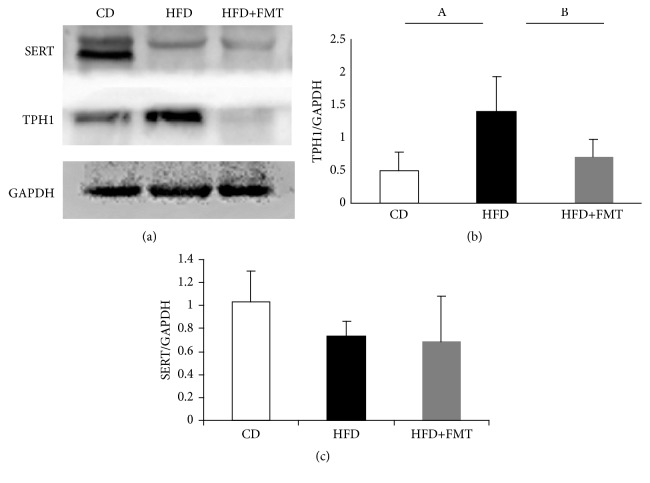
The protein levels of TPH1 and SERT in small intestine among the three groups. (a) The protein levels of TPH1 and SERT were measured using Western blot. (b) and (c) Quantitative analysis of TPH1 and SERT protein expression. A, p=0.009, compared with the control diet rats. B, p=0.029, compared with the HFD-fed rats receiving FMT.

The HFD-fed rats had a higher serotonin concentration in the serum and colon compared with the rats on the control diet by trend, and there were significant differences in the small intestine serotonin concentration between the HFD-fed rats and the controls (P = 0.020, Figure 2). The serotonin staining of the small intestine indicated a higher concentration of serotonin in the HFD-fed rats than in the controls (P = 0.012, Figure 3). The HFD-fed rats had significantly higher levels of TPH1 protein in the small intestine than the controls (P = 0.009, Figure 4). SERT protein expression in the small intestine was decreased, but not significant compared with the rats fed with the control diet (Figure 4).

### 3.3. Effect of FMT on the Intestinal Serotonin System

After receiving FMT, the serum, colon, and small intestine serotonin concentrations were decreased. There was a significant difference in the small intestine serotonin concentration between the FMT-treated HFD-fed rats and the nontreated HFD-fed rats (P = 0.030, Figure 2). The serotonin staining of the small intestine also showed a decreased concentration of serotonin in the HFD-fed rats after receiving FMT (Figure 3). The expression of TPH1 protein, which is the rate-limiting enzyme in the process of serotonin biosynthesis, was decreased in the HFD-fed rats after receiving FMT (P = 0.029, Figure 4), while no significant change of SERT expression was observed after receiving treatment (Figure 4).

### 3.4. Effect of FMT on Bile Acids in the Intestinal Contents

**Figure 5:**
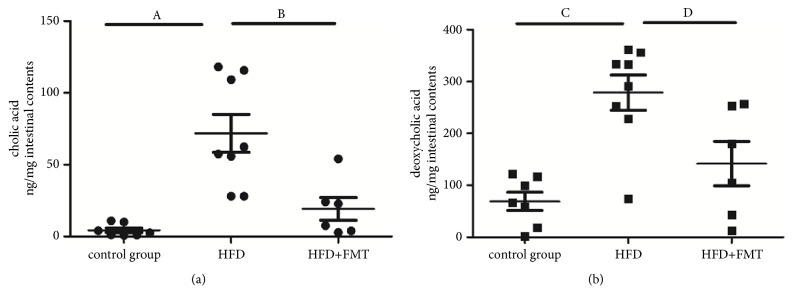
The levels of DCA and CA in intestinal contents of the three groups. (a) The CA levels of intestinal contents among the three groups (n=6~8). A, compared with control group, p<0.001; B, compared with HFD+FMT group, p=0.001. (b) The DCA levels of intestinal contents among the three groups (n=6~8). C, compared with control group, p<0.001; D, compared with HFD+FMT group, p=0.008.

Bile acid analysis indicated that the CA levels in the intestinal contents were higher in the HFD-fed rats than in the control group (P < 0.001, Figure 5(a)), and these levels decreased after receiving FMT treatment (P = 0.001, Figure 5(a)). The DCA levels were also higher in the HFD-fed rats than in the control group (P < 0.001, Figure 5(b)) and were decreased after receiving FMT (P = 0.008, Figure 5(b)). There were no significant differences in the levels of DCA and CA between the control group and the HFD + FMT group.

## 4. Discussion

This study showed that the HFD-fed rats had a faster small intestinal transit and increased serotonin synthesis in the small intestine compared with the control diet-fed rats. However, a 2-week intervention of FMT can alleviate the small intestinal transit and decrease the level of intestinal serotonin levels, along with a decrease in the concentration of the bile acids in the intestinal contents of the HFD-fed rats. This indicated that FMT may alleviate small intestinal transit in the HFD-fed rats. Thus, FMT may have a potential application in the treatment of gastrointestinal dysmotility in combination with obesity.

With obesity becoming increasingly prevalent, significant attention is being focused on obesity-related diseases. Obesity not only increases the risk of heart disease, NAFLD, and metabolic syndrome [33–35], but also is related to the alteration of gastrointestinal motility [7–9]. Our study showed that the gastrointestinal transit was faster in HFD-fed rats compared with that in rats fed a control diet. This result, which indicated that gastrointestinal dysmotility existed in obesity induced by HFD in the rats, was consistent with those of other studies that reported that the small bowel transit time was shorter in obese rats or HFD-fed mice [36, 37]. In addition, we found that gastrointestinal transit was faster in the HFD-fed rats than the control rats in this study. Moreover, the level of small intestinal serotonin was elevated in the HFD-fed rats compared with that in the rats on a standard diet. These results were consistent with those of previous studies [12, 28, 36]. Furthermore, the expression of TPH1 protein also increased, while the expression of SERT protein decreased in the HFD-fed rats. These results indicated that the changes in the TPH1 and SERT expression in the HFD-fed rats led to the increase in the small intestinal serotonin levels, which, in turn, regulated the gastrointestinal transit.

**Figure 6:**
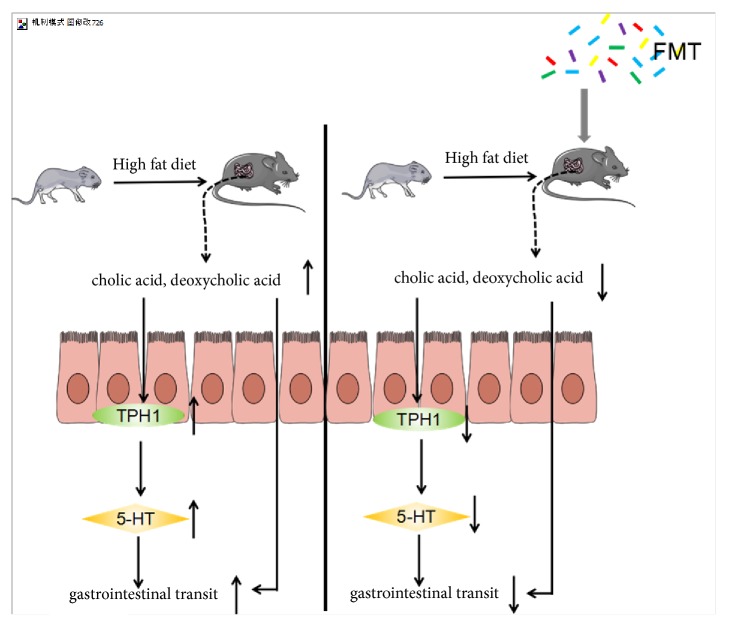
HFD could increase the levels of CA and DCA, leading to upregulating the expression of small intestinal TPH1 and then increase the serotonin concentration of small intestine, which fastens the gastrointestinal motility. Furthermore, after receiving FMT, the decreased levels of CA and DCA could downregulate the expression of TPH1 and reduce the serotonin concentration in the gut and then relieve the gastrointestinal dysmotility. In addition, DCA can also promote gastrointestinal motility.

Recently, an increasing number of studies support the association between gastrointestinal serotonin biosynthesis and gut microbiota [38, 39]. The expression of TPH1, a rate-limiting enzyme of serotonin synthesis in the gut, is regulated by metabolites of gut microbiota, such as DCA and CA [38, 39]. DCA, which is produced by microbial biotransformation of CA secreted by the liver [28], can upregulate the expression of TPH1 [38]; DCA can also enter into the circulatory system through the enterohepatic circulation and promote gastrointestinal motility by activating TGR5 G-protein-coupled receptors on ECs [40] (Figure 6). In this study, we found that the increase in the serotonin and TPH1 levels was accompanied by an increase in CA and DCA levels in the HFD-fed rats. These results were consistent with previous results, which suggested that a high-fat diet could result in the accumulation of CA and secondary bile acids [41, 42]. A high-fat diet could increase the levels of cholesterol in the liver, which is required for the synthesis of bile acids [43]. In contrast, a high-fat diet could induce the secretion of bile acids and their release into the gut. Previous studies have indicated that both CA and DCA could lead to the upregulation of the expression of TPH1 [38], and this may explain our results that the HFD-fed rats gastrointestinal transit was faster and the level of serotonin was higher in the small intestine in the HFD-fed rats than in the control diet rats.

At present, there is no consensus on an effective and optimal treatment for obesity and its related disorders. With mounting evidence supporting the association between obesity and gut microbiota [44], it is expected that gut microbiota-based therapy can reverse metabolic disorders, such as obesity and its related diseases [45]. In addition, a single-arm open-label population-based study showed that FMT is a safe and relatively effective treatment for IBS, in which gastrointestinal dysmotility is involved [26]. However, the underlying mechanism remains unknown. In this study, we found that those HFD-fed rats that received fecal microbiota from the control diet-fed rats presented a decreased gastrointestinal motility. After FMT for only two weeks, the TPH1 expression, and the concentration of serotonin in the small intestinal tissues reduced. Furthermore, our results showed that FMT was also able to reduce the DCA and CA concentration of the intestinal contents in the HFD-fed rats, which may be the factors that could downregulate TPH1 expression [38, 42]. This indicated that FMT could impact the biotransformation of the bile acids, and this may have a close association with the intestinal microbiota.

Our study, however, has the following limitations. We only conducted the quantification of CA and DCA of the intestinal contents. Whole metabolomic analysis and whole genome sequencing would be helpful to evaluate the effect of FMT on rats.

In summary, our study suggests that an HFD could increase the levels of CA and DCA, leading to upregulation of the expression of TPH1 in the small intestine, and an increase in the serotonin concentration, which, in turn, increases the gastrointestinal motility. Furthermore, after receiving FMT, the decreased levels of CA and DCA could downregulate the expression of TPH1 and reduce the serotonin concentration in the gut, thus, relieving gastrointestinal dysmotility (Figure 6). It provides a perspective on the association between obesity and gastrointestinal dysmotility, as well as on the necessity for gut microbiota-based treatment for the alleviation of gastrointestinal dysmotility.

## Figures and Tables

**Table 1:** Body weight, liver weight, ratio of liver weight and body weight, and gastrointestinal transit of rats from different diets at the end of the experiment. Means ± SEM are shown. Significant differences are indicated by small letters.

	**CD**	**HFD**	**HFD + FMT**
**Body weight (g)**	406.40 ± 26.36	489.80 ± 33.89^a^	466.67 ± 27.39^b^
**Liver weight (g)**	10.48 ± 0.81	18.46 ± 1.29^c^	16.36 ± 0.04^d^
**Liver weight: body weight (%)**	2.57 ± 0.68	3.77 ± 0.20^e^	3.51 ± 0.20^f^
**Length of intestine (cm)**	113.00 ± 10.82	100.00 ± 1.73	108.50 ± 14.08
**Small intestine motility (%)**	40.79 ± 5.99	52.03 ± 4.81^g,h^	41.75 ± 3.92

CD: control diet; HFD: high fat diet; FMT: fecal microbiota transplantation; a, compared with control diet rats, p=0.001; b compared with control diet rats, p=0.020; c, compared with control diet rats, p<0.001; d, compared with control diet rats, p=0.013; e, compared with control diet rats, p<0.001, compared with HFD + FMT group, p=0.050; f, compared with control diet rats, p<0.001; g, compared with control diet rats, p=0.033; h, compared with HFD + FMT group, p=0.045.

## Data Availability

The data used to support the findings of this study are available from the corresponding author upon request.
